# Determinants of developing cardiovascular disease risk with emphasis on type-2 diabetes and predictive modeling utilizing machine learning algorithms

**DOI:** 10.1097/MD.0000000000040813

**Published:** 2024-12-06

**Authors:** Shatabdi Das, Riaz Rahman, Ashis Talukder

**Affiliations:** a Science Engineering and Technology School, Khulna University, Khulna, Bangladesh; b Science Engineering and Technology School, Khulna University, Khulna, Bangladesh; c National Centre for Epidemiology and Population Health, Australian National University, Canberra, Australia.

**Keywords:** Bangladesh, cardiovascular diseases (CVD), diabetes, machine learning, risk factors

## Abstract

This research aims to enhance our comprehensive understanding of the influence of type-2 diabetes on the development of cardiovascular diseases (CVD) risk, its underlying determinants, and to construct precise predictive models capable of accurately assessing CVD risk within the context of Bangladesh. This study combined data from the 2011 and 2017 to 2018 Bangladesh Demographic and Health Surveys, focusing on individuals with hypertension. CVD development followed World Health Organization (WHO) guidelines. Eight machine learning algorithms (Support Vector Machine, Logistic Regression, Decision Tree, Random Forest, Naïve Bayes, K-Nearest Neighbor, Light GBM, and XGBoost) were analyzed and compared using 6 evaluation metrics to assess model performance. The study reveals that individuals aged 35 to 54 years, 55 to 69 years, and ≥ 70 years face higher CVD risk with adjusted odds ratios (AOR) of 2.140, 3.015, and 3.963, respectively, compared to those aged 18 to 34 years. “Rich” respondents show increased CVD risk (AOR = 1.370, *P* < .01) compared to “poor” individuals. Also, “normal weight” (AOR = 1.489, *P* < .01) and “overweight/obese” (AOR = 1.871, *P* < .01) individuals exhibit higher CVD risk than “underweight” individuals. The predictive models achieve impressive performance, with 75.21% accuracy and an 80.79% AUC, with Random Forest (RF) excelling in specificity at 76.96%. This research holds practical implications for targeted interventions based on identified significant factors, utilizing ML models for early detection and risk assessment, enhancing awareness and education, addressing urbanization-related lifestyle changes, improving healthcare infrastructure in rural areas, and implementing workplace interventions to mitigate stress and promote physical activity.

## 1. Introduction

Cardiovascular diseases (CVD) pose a significant global health challenge, ranking among the most pressing concerns worldwide. With its status as the primary cause of mortality on a global scale, CVD encompasses a range of heart and blood vessel conditions.^[1–3]^ This group of diseases stands as the leading cause of death in middle-aged and elderly populations, accounting for 1-third of all fatalities.^[4]^ Tragically, low-income and middle-income countries experience a disproportionately high burden of cardiovascular disease-related deaths.^[5]^ Moreover, the economic impact weighs heavily on patients and their families, imposing considerable financial strain.^[6,7]^ On a broader societal scale, CVD exact a significant economic cost,^[8]^ with estimates indicating a staggering global burden of $3.7 trillion between 2010 and 2015.^[3]^

CVD accounts for over 31% of global deaths.^[9]^ In Asian countries, heart diseases are responsible for 22% of total mortality.^[10]^ In Bangladesh, a staggering increase in cardiovascular disease-related deaths, with the number rising from 11 per 10,000 people in 1986 to 411 per 10,000 people in 2006.^[11]^ In 2018 alone, 0.256 million people lost their lives to cardiovascular disease in Bangladesh. Notably, cardiovascular disease stands as the leading cause of death in middle- and low-income nations, contributing to over 75% of all fatalities.^[12,13]^ According to a study published in January 2017, CVD has become the leading cause of death worldwide, climbing to the top of the list of the top 10 causes of death over the previous 15 years. In 2015 alone, it claimed 15 million lives.^[14]^ The World Health Organization (WHO) estimated that 17.5 million individuals died from CVD in 2005.^[1]^ By 2015, CVD had claimed the lives of approximately 17.9 million individuals.^[12,15]^ Each year, 12 million people succumb to cardiovascular disease, making it the primary cause of mortality in both developing and developed nations. The WHO further predicts a 24.5% increase in deaths by 2030.^[10,13]^ Among all CVDs, heart attacks and strokes account for 85% of deaths.^[5]^

Multiple factors, either directly or indirectly, contribute to CVD.^[2,11,13]^ High blood pressure, body mass index (BMI), smoking, diabetes, cholesterol levels, age, gender, stress, and family history all serve as risk factors for these disorders.^[2,4,11,13]^ Early detection and prediction play crucial roles in combating this disease, with counseling and medication forming the foundation of treatment when diagnosed early enough.^[2]^ Therefore, emphasis on the pre-detection of cardiovascular disease becomes paramount.^[10,12]^

Understanding the complex interplay between various risk factors and their effects on CVD is crucial for effective risk assessment and prevention strategies. Previous research has identified several established risk factors, such as hypertension, diabetes, obesity, and family history. However, there is still a need to explore research with advanced techniques to identify factors that may contribute to the development of CVD. Besides these factors it is highly necessary to identify a predictive model that has the ability of early prediction of the disease’s status. Machine learning (ML) algorithms offer a promising approach for predicting CVD risk with improved accuracy and precision. Numerous studies have demonstrated the potential of ML algorithms, including Support Vector Machine (SVM), Logistic Regression (LR), Decision Tree (DT), Random Forest (RF), Naïve Bayes (NB), K-Nearest Neighbor (KNN), LightGBM, XGBoost etc in detecting and aiding the early diagnosis of coronary artery disease (CAD), which is 1 of the leading causes of mortality worldwide.^[16]^ For example, Garavand et al highlighted that SVM and RF algorithms achieved the highest accuracy rates in diagnosing CAD, showcasing their utility in supporting clinical decision-making.^[17]^ Additionally, research by Komal Kumar et al emphasized the effectiveness of various ML models for cardiovascular disease risk prediction, suggesting that leveraging multiple approaches can lead to improved patient outcomes.^[18]^ Alizadehsani and colleagues conducted a comprehensive review of ML techniques in CAD detection, illustrating the breadth of applications and benefits that these technologies can offer.^[19]^ Collectively, these findings advocate for the integration of advanced ML algorithms into clinical practice to enhance diagnostic precision and facilitate timely interventions. These algorithms can handle large and complex datasets, identify non-linear relationships, and discover patterns that may not be apparent through conventional statistical methods. Therefore, this manuscript aims to investigate the factors associated with the high-risk of developing cardiovascular disease and explore the feasibility of predicting CVD using ML algorithms. Through the exploration of a wide array of variables and the utilization of advanced computational techniques, this research aims to enhance our comprehensive understanding of the influence of type-2 diabetes on the development of CVD risk, its underlying determinants, and to construct precise predictive models capable of accurately assessing CVD risk within the context of Bangladesh.

## 2. Methods

### 2.1. Sources of data

Our research data extracted from 2 distinct surveys: the 2011 Bangladesh Demographic and Health Survey (BDHS) and the 2017 to 2018 BDHS. In 2011 BDHS, a cohort of 1154 individuals and in 2017 to 2018 BDHS 2973 individuals who had hypertension were used with no missing data points. By combining and merging the datasets from both surveys, we had a comprehensive dataset comprising a total of 4127. The process of combining data sets is shown in Table 1. The data is freely available in the following link: https://dhsprogram.com/data/available-datasets.cfm.

**Table 1:** Combining the data sets.

Surveys	No. of samples	No. samples with hypertension	No. samples with hypertension and without missing values
BDHS-2011	83,731	1790	1154
BDHS-2017 to 2018	89,819	3661	2973
Total	1,73,550	5451	4127

### 2.2. Sample design and sampling frame

Starting in 1984, the DHS Program provided technical support to conduct more than 300 demographic and health surveys across more than 90 countries. These surveys employ a meticulously designed sampling methodology. The process begins with a stratified multistage cluster design overseen by the DHS. In the initial phase, probability proportional to size (PPS) is applied to choose the principal sample units (PSUs) within each stratum. Subsequently, in the second phase, an exhaustive inventory of households is compiled for each selected cluster. Within these chosen clusters, households are then systematically sampled with equal probability to ensure a representative and robust data collection process.

### 2.3. Variable description

#### 2.3.1. Dependent variable

In this research, our dependent variable of interest is the “Development of CVD Risk.” To construct this variable, we have derived it from a combination of factors related to hypertensive patients and diabetes. We have substantially reorganized the data originally presented in the 1999 WHO/ISH recommendations to delineate 3 primary risk groups for significant cardiovascular events expected to occur within the next decade among individuals with hypertension.^[4]^ Initially, we focus on hypertensive individuals with systolic blood pressure (SBP) ≥ 140 or diastolic blood pressure (DBP) ≥ 90, categorizing them into 3 distinct stages: Stage 1 (SBP 140-159 or DBP 90-99), Stage 2 (SBP 160-179 or DBP 100-109), and Stage 3 (SBP ˃ 180 or DBP ˃ 110). In addition, we consider the type-2 diabetes (T2D) status of these individuals, where those with a fasting plasma blood glucose level ≥ 7.0 mmol/L are classified as having diabetes, while those with levels below this threshold are categorized as not having diabetes. Based on the presence or absence of diabetes, we further classify hypertensive patients into 3 categories: low-risk, medium risk, and high-risk. This composite variable, referred to as the “Development of CVD Risk,” encompasses these 3 distinct risk categories and serves as the primary focus of our study (Table 2).

**Table 2:** Measuring variable with blood pressure and diabetes.

Blood pressure (mm Hg)			
Type-2 diabetes status	Stage 1	Stage 2	Stage 3
	SBP 140 to 159 or DBP 90 to 99	SBP 160 to 179 or DBP 100 to 109	SBP >180 or DBP >110
Absent	Low	Medium	High
Present	Medium	High	High

For our analysis, we pinpointed individuals at a higher risk of developing CVD. This high-risk group includes those with a previous history of diabetes and/or those falling into the higher blood pressure categories (Stage 2 or Stage 3). In other words, individuals meeting these criteria are considered to be at high-risk for CVD. That is

$$\text{High risk of developing CVD}=\{\begin{array}{cc} 1; & \begin{array}{c} \text{Yes;}\;\text{if T2D present and/or }\\ \text{fall into the Stage 2 or 3}\; \end{array}\\ 0; & \text{Otherwise } \end{array}$$

#### 2.3.2. Independent variables

The independent variables in our study encompass a set of 10 attributes: division, gender, employment status, age, education, wealth index, residential location, marital status, household size, and BMI. Notably, the 2011 BDHS featured 7 divisions: Barisal, Chittagong, Dhaka, Khulna, Rajshahi, Rangpur, and Sylhet. In contrast, the 2017 to 2018 BDHS data included 8 divisions, with the addition of Mymensingh. It is worth noting that Dhaka and Mymensingh divisions were merged on September 14, 2015, resulting in 7 categories for our analysis. We also categorized the wealth index into 5 groups: lowest, poorer, medium, richer, and wealthiest. To simplify our analysis, we combined the poorest and poorer categories into “poor,” designated the medium category as “middle,” and retained the wealthiest category as “wealthy.”

### 2.4. Data pre-processing

#### 2.4.1. Data cleaning

Upon combining the data from the 2011 BDHS and the 2017 to 2018 BDHS, we initially had a dataset comprising 21,959 respondents. However, not all observations contained complete information. Specifically, out of these, 914 samples provided comprehensive data pertaining to the development of CVD and other relevant features. Following a rigorous process of data cleaning to address missing values, we retained a final dataset containing 914 complete observations.

#### 2.4.2. Data balancing

Imbalance in class distribution has been a persistent challenge for researchers in various ML applications, adversely impacting accuracy.^[2]^ When the number of samples varies significantly across different classes, it poses a significant issue. To address this, a common approach involves balancing the underrepresented class in the dataset.^[20]^ In this study, we employed the Synthetic Minority Oversampling Technique (SMOTE) to rectify this class imbalance issue. Following the balancing of the dataset, all subsequent analyses were conducted using this balanced dataset, wherein various ML algorithms were applied for the classification of CVD development.

### 2.5. Statistical analysis and ML models

After filtering out missing, ineligible, and non-responsive cases for the questions, we scrutinized the completeness of the extracted data. Initially, we conducted univariate analysis and assessed the bivariate associations between independent and dependent variables using the chi-square test. Subsequently, employing multivariable binary LRs, we explored the impact of other factors. To address the complexity of the sample design, we applied sample weights provided with the data to all our analyses. All statistical analyses were conducted using MS-Excel and SPSS Windows version 25.0 on the remaining dataset. The outcomes are presented in tabular form.

Following this, we employed a variety of ML models, including SVM, DT, RF, NB, KNN, LightGBM, and

Boost, to identify the most accurate model for predicting CVD risk. The performance of these ML models was evaluated using various metrics such as Accuracy, Precision, Sensitivity, Specificity, F1 score, and Area Under the Curve (AUC) values.

## 3. Results

Table 3 provides a comprehensive overview of the data from BDHS-2011, BDHS-2017 to 2018, and the combined dataset, offering insights into the socio-demographic characteristics of the respondents. As depicted in Table 3, noteworthy findings emerged. In the BDHS-2011 data, the Khulna division exhibited the highest representation, accounting for 20.5% of the sample, while the Sylhet division had the lowest frequency at 9.7%. Among the respondents, males constituted the majority, comprising 53.2% of the total. A significant proportion of respondents, approximately 47.8%, had no formal education or were in the preschool category. Furthermore, a substantial portion, 57.2%, were not actively employed. The highest frequency of respondents, at 38.7%, was observed among those with 1 to 4 household members. In terms of age distribution, the 55 to 69 years age group stood out with the highest frequency, encompassing 40.0% of the respondents. Those categorized as “rich” based on wealth index accounted for the largest share, representing 53.7% of the dataset. Additionally, rural areas were predominant, with 62.0% of the respondents residing there. The highest frequency, at 71.3%, was recorded among respondents who were currently married. In relation to body weight, the category of “normal-weighted” respondents held the highest frequency at 58.0%, while the category of respondents with a low-risk of developing CVD had the highest frequency at 77.7%.

**Table 3:** Descriptive statistics of the BDSH-2011, BDHS-2017 to 18, and combined data.

Variables	Categories	BDHS-2011	BDHS-2017 to 2018	Combined data
		Total (%)	Total (%)	Total (%)
Division	Barisal	113 (9.8)	351 (11.8)	464 (11.2)
	Chittagong	139 (12.0)	410 (13.8)	549 (13.3)
	Dhaka	199 (17.2)	595 (20.0)	794 (19.2)
	Khulna	236 (20.5)	452 (15.2)	688 (16.7)
	Rajshahi	150 (13.0)	410 (13.8)	560 (13.6)
	Rangpur	205 (17.8)	452 (15.2)	657 (15.9)
	Sylhet	112 (9.7)	303 (10.2)	415 (10.1)
Sex of household member	Male	614 (53.2)	1272 (42.8)	1886 (45.7)
	Female	540 (46.8)	1701 (57.2)	2241 (54.3)
Highest education level attained	No education	552 (47.8)	966 (32.5)	1518 (36.8)
	Primary	276 (23.9)	866 (29.1)	1142 (27.7)
	Secondary	195 (16.9)	725 (24.4)	920 (22.3)
	Higher	131 (11.4)	416 (14.0)	547 (13.3)
Work Status	No	660 (57.2)	1246 (41.9)	1906 (46.2)
	Yes	494 (42.8)	1727 (58.1)	2221 (53.8)
Number of household member	1 to 4	447 (38.7)	1298 (43.7)	1745 (42.3)
	5 to 6	395 (34.2)	973 (32.7)	1368 (33.1)
	≥7	312 (27.0)	702 (23.6)	1014 (24.6)
Age	18 to 34 yr	Null	618 (20.8)	618 (15.0)
	35 to 54 yr	418 (36.2)	1209 (40.7)	1627 (39.4)
	55 to 69 yr	462 (40.0)	754 (25.4)	1216 (29.5)
	≥70 yr	274 (23.7)	392 (13.2)	666 (16.1)
Wealth index	Poor	326 (28.2)	1010 (34.0)	1336 (32.4)
	Middle	208 (18.0)	581 (19.5)	789 (19.1)
	Rich	620 (53.7)	1382 (46.5)	2002 (48.5)
Place of residence	Rural	716 (62.0)	1881 (63.3)	2597 (62.9)
	Urban	438 (38.0)	1092 (36.7)	1530 (37.1)
Marital status	Never married/divorced/separated/widow	331 (28.7)	661 (22.2)	992 (24.0)
	Currently married	823 (71.3)	2312 (77.8)	3135 (76.0)
Body mass index (BMI)	Underweight	275 (23.8)	348 (11.7)	623 (15.1)
	Normal	669 (58.0)	1545 (52.0)	2214 (53.6)
	Overweight/obese	210 (18.2)	1080 (36.3)	1290 (31.3)
High risk of developing CVD	No	897 (77.7)	2285 (76.9)	3182 (77.1)
	Yes	257 (22.3)	688 (23.1)	945 (22.9)

Abbreviation: CVD = cardiovascular diseases.

In the BDHS-2017 to 2018 dataset, as presented in Table 3, the Dhaka division exhibited the highest representation, constituting 20.0% of the sample, whereas Sylhet had the lowest frequency at 10.2%. Female respondents accounted for the majority, comprising 57.2% of the dataset. The largest frequency among respondents was observed in the category with no formal education or preschool background, accounting for 32.5%. A significant proportion of respondents, approximately 58.1%, were currently employed. In terms of household size, those with 1 to 4 household members had the highest frequency at 43.7%. The age group ranging from 35 to 54 years represented the largest share, with 40.7% of respondents falling into this category. Additionally, a notable 46.5% were classified under the “rich” category based on their wealth index. Rural areas were predominant, with 63.3% of respondents residing there. Furthermore, the highest frequency, at 71.8%, was recorded among respondents who were currently married. Concerning body weight, “normal-weighted” respondents held the highest frequency at 52.0%, while “underweight” respondents had the lowest frequency at 11.7%. Among respondents, those with the lowest risk of developing CVD exhibited the highest frequency, encompassing 76.9% of the sample.

In the combined dataset, as illustrated in Table 3, the Sylhet division displayed the lowest frequency at 10.1%, whereas the Dhaka division exhibited the highest representation at 19.2%. Females constituted the majority of respondents, accounting for 54.3% of the dataset. Among respondents, the highest frequency was observed in the category reporting no formal education or preschool experience, standing at 36.8%. A significant proportion, approximately 53.8%, were currently employed. In terms of household size, the largest frequency was found among those with 1 to 4 household members, representing 42.3% of the sample. The age group spanning 35 to 54 years had the highest frequency, with 39.4% of respondents falling into this category. Furthermore, 48.5% of the population fell under the high wealth index classification. Rural areas were predominant, with 62.9% of respondents residing there. Additionally, the highest frequency, at 76.0%, was reported among respondents who were currently married. In relation to body weight, respondents categorized as “normal-weighted” had the highest frequency, accounting for 53.6%. Conversely, those with a low-risk of developing cardiovascular disease had the highest frequency, which was 77.1%.

Table 4 shows the bivariate associations between various socio-demographic and health-related variables and the risk of developing CVD. Significant associations (*P* < .05) are observed for division (χ² = 13.622, *P* < .05), work status (χ² = 8.212, *P* < .01), age (χ² = 62.839, *P* < .01), wealth index (χ² = 36.153, *P* < .01), place of residence (χ² = 11.734, *P* < .01), and BMI (χ² = 19.913, *P* < .01). Dhaka division has the highest proportion of individuals at high CVD risk, while Sylhet has the lowest. Those who are not working have a slightly higher risk than working individuals. Age shows a strong association, with older age groups displaying progressively higher CVD risk, especially those aged 70 and above. Wealthier individuals are also at greater risk, with 13% of the “rich” group classified as high-risk, compared to 5.9% of the “poor.” Rural residents face a higher risk than urban dwellers, and overweight/obese individuals have an elevated risk compared to those with normal or low BMI. In contrast, sex of household head (χ² = 0.777, *P* = .378), education level (χ² = 2.037, *P* = .565), household size (χ² = 1.416, *P* = .493), and marital status (χ² = 2.135, *P* = .144) do not show significant associations with CVD risk in this dataset.

**Table 4:** Bivariate association in combined dataset.

Variables	High-risk of developing CVD		$\chi^{\text{2}}$	*P* value
	No (%)	Yes (%)		
Division				
Barisal	364 (8.8)	100 (2.4)	13.622	<.05
Chittagong	405 (9.8)	144 (3.5)		
Dhaka	600 (14.5)	194 (4.7)		
Khulna	522 (12.6)	166 (4.0)		
Rajshahi	432 (10.5)	128 (3.1)		
Rangpur	537 (13.0)	120 (2.9)		
Sylhet	322 (7.8)	93 (2.3)		
Sex of household member				
Male	1466 (35.5)	420 (10.2)	0.777	.378
Female	1716 (41.6)	525 (12.7)		
Highest education level attained				
No education, preschool	1166 (28.3)	352 (8.5)	2.037	.565
Primary	897 (21.7)	245 (5.9)		
Secondary	700 (17.0)	220 (5.3)		
Higher	419 (10.2)	128 (3.1)		
Work status				
No	1431 (34.7)	475 (11.5)	8.212	<.01
Yes	1751 (42.4)	470 (11.4)		
Number of household members				
1 to 4	1352 (32.8)	393 (9.5)	1.416	.493
5 to 6	1040 (25.2)	328 (7.9)		
≥7	790 (19.1)	224 (5.4)		
Age				
18 to 34 yr	542 (13.1)	76 (1.8)	62.839	<.01
35 to 54 yr	1270 (30.8)	357 (8.7)		
55 to 69 yr	900 (21.8)	316 (7.7)		
≥70 yr	470 (11.4)	196 (4.7)		
Wealth index				
Poor	1091 (26.4)	245 (5.9)	36.153	<.01
Middle	627 (15.2)	162 (3.9)		
Rich	1464 (35.5)	538 (13)		
Place of residence				
Rural	2047 (49.6)	550 (13.3)	11.734	<.01
Urban	1135 (27.5)	395 (9.6)		
Marital status				
Never married/divorced/separated/widow	748 (18.1)	244 (5.9)	2.135	.144
Currently married	2434 (59.0)	701 (17.0)		
Body mass index (BMI)				
Underweight	515 (12.5)	108 (2.6)	19.913	<.01
Normal	1717 (41.6)	497 (12.0)		
Overweight/obese	950 (23.0)	340 (8.2)		

Abbreviation: CVD = cardiovascular diseases.

Table 5 presents the results of binary LR analysis conducted exclusively with the combined dataset. The table reveals that all age categories yield statistically significant results (P≤.05) . Specifically, respondents aged 35 to 54 years, 55 to 69 years, and those above 70 years are 2.140 times, 3.015 times, and 3.963 times more likely, respectively, to exhibit a high-risk of developing CVD compared to respondents aged 18 to 34 years. Regarding the wealth index, affluent respondents are 1.370 times more likely (AOR=1.370, P≤.01) to face a high-risk of developing CVD in comparison to their less affluent counterparts. Additionally, the likelihood of having a high-risk for developing CVD is 1.489 times higher for respondents with a normal weight and 1.871 times higher for those categorized as overweight or obese, as opposed to respondents classified as underweight. Notably, the variable of BMI exhibits statistically significant results across all categories (P≤.05) .

**Table 5:** Logistic regression model showing factors affecting the development of cardiovascular diseases in combined data.

Variable	Categories	AOR	*P* value	95% CI for OR	
				Lower	Upper
Division	Barisal (ref)				
	Chittagong	1.235	0.170	0.914	1.670
	Dhaka	1.129	0.401	0.851	1.497
	Khulna	1.060	0.694	0.793	1.417
	Rajshahi	1.099	0.541	0.811	1.490
	Rangpur	0.876	0.395	0.646	1.188
	Sylhet	1.098	0.580	0.789	1.527
Sex of household member	Male (ref)				
	Female	1.143	0.184	0.938	1.392
Highest education level attained	No education, preschool (ref)				
	Primary	0.992	0.934	0.811	1.213
	Secondary	1.180	0.161	0.936	1.487
	Higher	1.139	0.380	0.852	1.522
Work status	No (ref)				
	Yes	1.002	0.982	0.827	1.215
Number of household members	1 to 4 (ref)				
	5 to 6	1.030	0.741	0.865	1.226
	≥7	.856	0.122	0.702	1.043
Age	18 to 34 yr (ref)				
	35 to 54 yr	2.140	<0.01	1.619	2.829
	55 to 69 yr	3.015	<0.01	2.247	4.047
	≥70 yr	3.963	<0.01	2.838	5.535
Wealth index	Poor (ref)				
	Middle	1.074	0.542	0.854	1.351
	Rich	1.370	<0.01	1.111	1.690
Place of residence	Rural (ref)				
	Urban	1.111	0.215	0.941	1.312
Marital status	Never married/divorced/separated/widow (ref)				
	Currently married	0.962	0.715	0.783	1.183
Body mass index (BMI)	Underweight (ref)				
	Normal	1.489	<0.01	1.170	1.896
	Overweight/Obese	1.871	<0.01	1.429	2.451

Abbreviations: AOR = adjusted odds ratios, CVD = cardiovascular diseases, Ref = Reference category.

Table 6 provides a comprehensive evaluation of selected algorithms used to assess classification performance. The findings in Table 6 indicate that the RF model consistently excels, achieving the highest accuracy rate at 75.21%, precision at 75.38%, sensitivity at 73.08%, F1 score at 75.19%, and an AUC value of 80.79%. In terms of specificity, the SVM model stands out, boasting the highest value at 77.75%. Consequently, when considering the overall classification results, the RF model emerges as the most effective algorithm for predicting the onset of cardiovascular disease.

**Table 6:** Classification performance measure of the algorithms and comparison.

Algorithms	Accuracy	Precision	Sensitivity	Specificity	F1 score	AUC
SVM	73.38%	73.43%	68.68%	77.75%	73.31%	75.65%
DT	72.31%	72.42%	69.66%	74.78%	72.28%	74.37%
RF	75.21%	75.38%	73.08%	76.96%	75.19%	80.79%
LightGBM	70.58%	70.66%	68.64%	72.59%	70.58%	74.74%
XGBoost	74.35%	74.35%	71.81%	76.62%	74.33%	80.28%
LR	60.02%	60.03%	59.56%	60.46%	60.02%	64.74%
KNN	71.01%	71.39%	59.43%	60.59%	70.82%	79.16%
NB	60.34%	60.44%	56.53%	62.04%	60.29%	63.46%

Abbreviations: AUC = area under the curve, DT = decision tree, KNN = K-nearest neighbors, LightGBM = light gradient boosting machine, LR = logistic regression, NB = Naïve Bayes, RF = random forest, SVM = support vector machine, XGBoost = extreme gradient boosting.

### 3.1. 10-Fold cross validation of the accuracy of the classifiers

Table 7 presents the average classification accuracy resulting from 10-fold cross validation for all ML models. The table’s findings reveal that the RF model achieved the highest average accuracy score, reaching 71%. In contrast, both SVM and XGBoost models obtained the same accuracy score, which stood at 69%. Additionally, the DT and KNN models demonstrated identical average accuracy rates of 68%. Moreover, LightGBM, LR, and NB models exhibited average accuracy scores of 67%, 59%, and 58%, respectively. Notably, among the selected models, NB yielded the lowest average accuracy.

**Table 7:** 10-Fold cross validation scores.

Fold	ML classifier							
	SVM	DT	RF	LightGBM	XGBoost	LR	KNN	NB
Fold-01	0.74	0.68	0.70	0.67	0.70	0.60	0.69	0.54
Fold-02	0.68	0.67	0.69	0.69	0.67	0.57	0.69	0.59
Fold-03	0.72	0.70	0.75	0.67	0.72	0.60	0.70	0.57
Fold-04	0.61	0.63	0.66	0.68	0.71	0.64	0.67	0.61
Fold-05	0.65	0.67	0.70	0.64	0.68	0.57	0.68	0.56
Fold-06	0.71	0.69	0.74	0.65	0.68	0.57	0.69	0.61
Fold-07	0.71	0.68	0.72	0.68	0.67	0.56	0.62	0.57
Fold-08	0.68	0.69	0.73	0.64	0.67	0.63	0.68	0.57
Fold-09	0.69	0.68	0.71	0.66	0.74	0.58	0.69	0.59
Fold-10	0.66	0.67	0.71	0.68	0.71	0.58	0.66	0.59
Average	0.69	0.68	0.71	0.67	0.69	0.59	0.68	0.58

Abbreviation: ML = machine learning.

**Figure 1.**
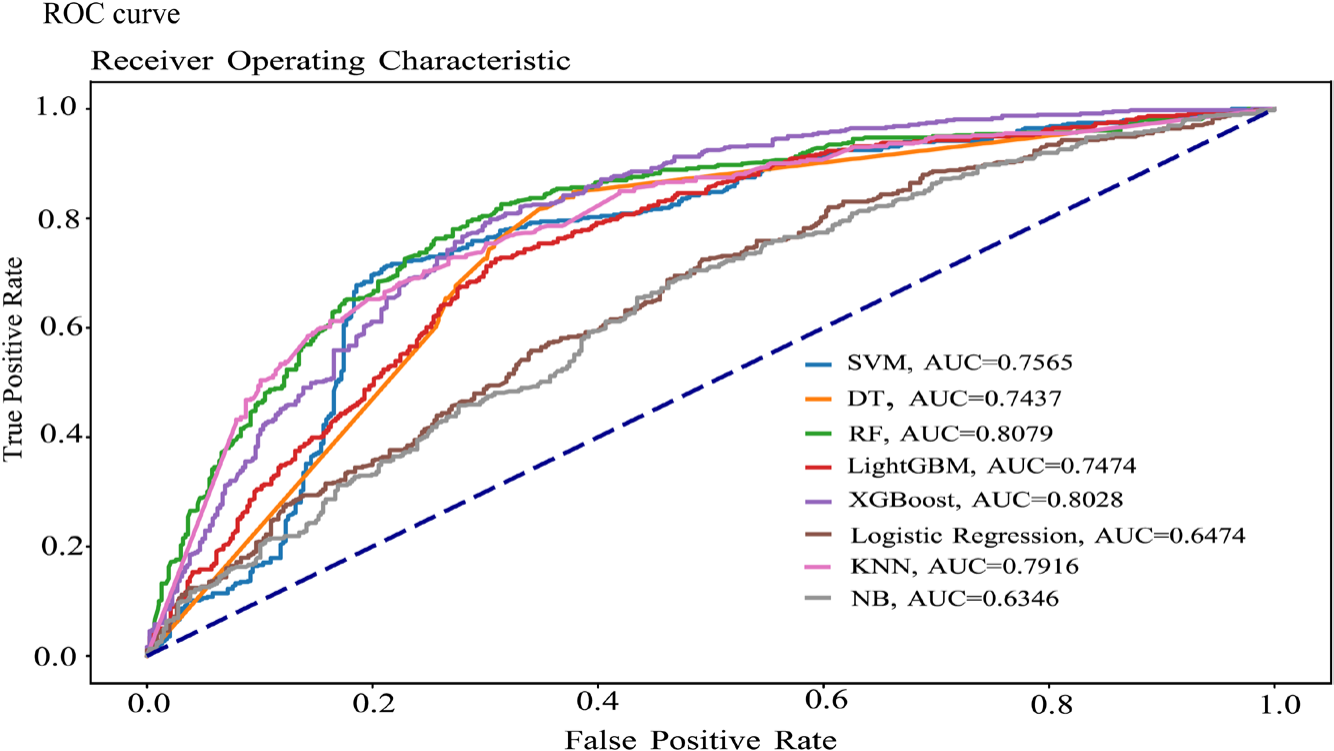
ROC curve for all selected model. ROC = receiver operating characteristic.

Figure 1 visually depicts the classification performance of all classifiers (SVM, DT, RF, LightGBM, XGBoost, LR, KNN, NB) through receiver operating curves (ROC) and their corresponding AUC values. The ROC curve serves as a competency measurement plot for any classifier, representing the trade-off between true positive rate (sensitivity) and false positive rate (1-specificity) for object classification. Different points on the curve correspond to various decision thresholds used to classify objects as positive or negative, revealing the optimal balance between sensitivity and false positive rate. The results from Figure 1 indicate that the RF classifier achieved the highest AUC value at 80.79%, outperforming all other classifier models. The XGBoost model secured the second-highest AUC value at 80.28%. Notably, all other classifiers achieved AUC values exceeding 70%, with the exceptions being LR and the NB classifier, both of which scored below 65%.

**Figure 2.**
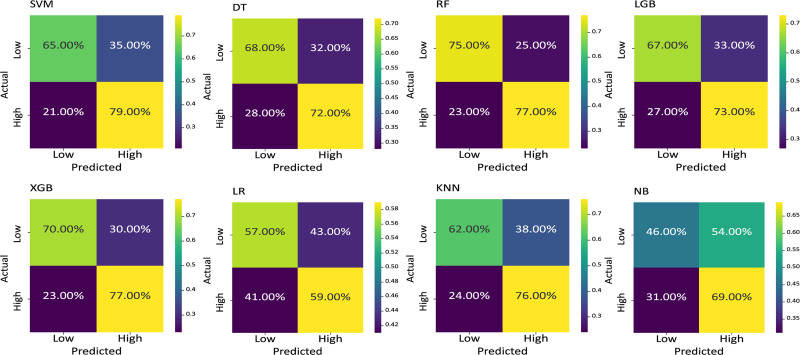
Confusion matrix of the models.

Based on the results in confusion matrix in Figure 2, we can notice specific differences in performance. For the Support SVM, RF, and XGBoost models, there is a higher rate of correctly classified cases, where RF achieve the best balance of accuracy. Explicitly, RF shows 75.0% accuracy in predicting low-risk cases and 77.0% for high-risk cases, which makes it the top-performing model in terms of correctly identifying both risk cases. The DT and LightGBM models show marginally lower performance, with accuracy percentages ranging in the low 70s, and LR and NB demonstrate comparatively weaker performance, especially in predicting low-risk cases.

Therefore, RF has the highest effectiveness in predicting both Low and High CVD risk, that’s why it is the most reliable model among the 8 tested.

## 4. Discussion

Cardiovascular disease, a highly heritable trait, causes major deaths worldwide. Though the prevalence of CVD has been found high worldwide, the awareness rate has been found very low.^[21]^ CVD is considered highly heritable trait, but the micronutrients intake, age, socio-economic condition, and environmental toxic metal condition can also cause severe risk of CVD.^[22–24]^ Throughout our study, we have tried to identify the risk factors and predict CVD using different ML models. The hypertensive patients from BDHS-2011 and BDHS-2017 to 2018 datasets have been used throughout the study for analysis.

Several recent studies have used ML algorithms to predict CVD which indicate the reliability and the feasibility of this method in this case.^[25–27]^ Chandralekha & Shenbagavadivu compared supervised and unsupervised ML models and found that DT has more classification accuracy, and precision with 73%, and 91% respectively. Another study conducted by Arunachalam found SVM and Multilayer Perceptron with the highest accuracy score (91.7%). It also identified chest pain type, thalassemia, age, depression, cholesterol, gender, blood pressure as the most effective factors for CVD. In addition, there was a study where they found out SVM and RF were most effective algorithms in their study with F-score 88% and 87% respectively.^[16]^

In this study, some statistical analysis such as frequency distribution and chi-square test were conducted to identify the patterns and also the significant factors. A slight increment of CVD was found from BDHS-2011 to BDHS-2017 to 2018 with the prevalence of 22.3% and 23.1% respectively. Chi-square analysis determined division, work status, age, wealth index, place of residence and BMI as significant factors. Besides statistical analysis, 8 different ML classifier models were used to predict CVD. Among these, RF was identified with the highest accuracy, precision, sensitivity, and F1 score with 78%, 78%, 74%, and 78% respectively. The features division, age, highest education level, BMI, wealth index, place of residence and work status has been identified as most important.

The highest prevalence of CVD had been occurred in Dhaka that might be the result of rapid urbanization, dietary changes, increased consumption of tobacco, limited physical activity, low level of awareness, and also the poor detection and control rate.^[28]^ This study has also shown that BMI is also working as a significant factor for CVD because obesity irritates plaque in the arteries and predisposes, releases substances in the blood that make plaque rapture, and also develops atrial fibrillation, increases triglyceride levels which triggers heart attacks, plaque rupturing, and stevens notes.^[29]^ Moreover, age is also an important feature for CVD, since it has been linked to obesity, persistent inflammation, and oxidative stress which may increase the risk of heart diseases.^[30]^ Our study has also been found that the prevalence of CVD is higher in rural areas that may happen because of the low level of awareness among people and also the inadequate health qualities.^[31]^ Another important risk factor determined by our study is wealth index which has also been found positively correlated with CVD. This may happen because of the accessibility of high-calories food from well-off families and also related with less physical activities.^[32]^

Working status has also been found positively correlated with CVD that means that less physical activity as well as intaking high-calories food and also stress may increase the risk of CVD.^[33]^ Moreover, the ML models determined education level as the most important significant factors for CVD. This explains the fact that low education may lead to low awareness and knowledge of healthy lifestyle, and also the risk of CVD.^[34]^

The findings of this study provide valuable insights and practical implications for addressing CVD. The statistical analysis identified several significant factors associated with CVD, including division, work status, age, wealth index, place of residence, and BMI. These factors can help healthcare professionals and policymakers prioritize interventions and allocate resources effectively.

The study also employed ML models, with RF achieving the highest accuracy, precision, sensitivity, and F1 score for predicting CVD. This suggests that ML models can be utilized as a reliable tool for early detection and risk assessment of CVD. The identified important features, such as division, age, highest education level, BMI, wealth index, place of residence, and work status, can guide the development of targeted interventions. For example, focusing on urban areas like Dhaka, where a higher prevalence of CVD was observed, interventions can address factors like rapid urbanization, dietary changes, increased tobacco consumption, limited physical activity, low awareness, and inadequate detection and control rates.

Promoting awareness and education about healthy lifestyles, especially among individuals with lower education levels, can help mitigate the risk of CVD. Targeted interventions in rural areas, aiming to improve health infrastructure and increase awareness, can contribute to reducing the burden of CVD in those communities. Addressing the correlation between wealth index and CVD requires strategies to promote healthy eating habits and physical activity among all socio-economic groups. Workplace interventions focusing on reducing stress and promoting physical activity can also contribute to preventing CVD.

## 5. Limitations

Since there was a significant gap between the 2 BDHS datasets that were combined, this may have influenced the results. Respondents related to the topic were very limited, for which the sample size is very small. Fasting plasma glucose (FPG) readings are used to monitor diabetes in BDHS, but they do not constitute a clinical diagnosis of the disease because, according to the WHO, “FPG alone cannot be used to diagnose diabetes, as it fails to diagnose around 30% of cases of previously undiagnosed diabetes. However, there is still room for improvement in the method that is currently being used.

## 6. Conclusion

In literature, numerous research disclose many classification techniques to identify the better diagnosis for CVD but the performance of the classifier is still inconsistent and none of the research was done based on Bangladesh. So, the aim of this study is to improve the literature with suggested classification techniques that yield a classifier with better accuracy for predicting the development of CVD in Bangladesh. Eight ML algorithms were used to predict the development of CVD and compared. For the measures of the classification performance, 6 types of evaluating measure were used such as accuracy, precision, sensitivity, specificity, F1 score and AUC value. I demonstrated my analysis by showing accuracy at 78% and Area Under Curve (AUC) at 84% through RF classifier. For specificity, KNN outperformed other algorithms. This concept has the potential to revolutionize the medical industry. By using this technique, it may be possible to identify heart disease-at-risk patients quickly, potentially reducing the rising death rate. Future advancements in ML algorithms will lead to a rise in the prevalence of this type of diagnosis. The model might be improved and modified if more patient data is used. Adapting this approach to other types of datasets will be intriguing in the future, as it could provide a time and money saving option for cardiovascular patients and doctors alike.

## Author contributions

**Conceptualization:** Shatabdi Das, Riaz Rahman, Ashis Talukder.

**Data curation:** Shatabdi Das, Ashis Talukder.

**Formal analysis:** Shatabdi Das, Riaz Rahman.

**Funding acquisition:** Ashis Talukder.

**Methodology:** Shatabdi Das, Riaz Rahman.

**Software:** Shatabdi Das, Riaz Rahman, Ashis Talukder.

**Supervision:** Ashis Talukder.

**Validation:** Shatabdi Das, Ashis Talukder.

**Writing—original draft:** Shatabdi Das, Riaz Rahman, Ashis Talukder.

**Writing—review & editing:** Shatabdi Das, Riaz Rahman, Ashis Talukder.
